# Is an 8 mm cutoff necessary when performing primary common bile duct closure after laparoscopic common bile duct exploration?

**DOI:** 10.12669/pjms.40.11.9441

**Published:** 2024-12

**Authors:** Danfeng Shen, Peng Chang, Haibin Xu, Hongxing Xu, Yingchao Lu

**Affiliations:** 1 Danfeng Shen, Department of Hepatobiliary Surgery, Taicang Affiliated Hospital of Soochow University, (The First People’s Hospital of Taicang), Suzhou 215400, Jiangsu, China; 2 Peng Chang, Department of Hepatobiliary Surgery, Taicang Affiliated Hospital of Soochow University, (The First People’s Hospital of Taicang), Suzhou 215400, Jiangsu, China; 3 Haibin Xu, Department of Hepatobiliary Surgery, Taicang Affiliated Hospital of Soochow University, (The First People’s Hospital of Taicang), Suzhou 215400, Jiangsu, China; 4 Hongxing Xu, Department of Hepatobiliary Surgery, Taicang Affiliated Hospital of Soochow University, (The First People’s Hospital of Taicang), Suzhou 215400, Jiangsu, China; 5 Yingchao Lu, Department of Hepatobiliary Surgery, Taicang Affiliated Hospital of Soochow University, (The First People’s Hospital of Taicang), Suzhou 215400, Jiangsu, China

**Keywords:** Laparoscopic common bile duct exploration, Primary duct closure, Common bile duct stone, Acute cholangitis

## Abstract

**Objective:**

To evaluate whether the efficacy and safety of primary duct closure(PDC) was limited to patients with a wider common bile duct(CBD) (≥8 mm).

**Methods:**

A retrospective study of patients who underwent PDC after laparoscopic common bile duct exploration (LCBDE) at Taicang Affiliated Hospital of Soochow University from March 2012 to June 2019 was performed. Fifty-five patients were enrolled and classified into two groups according to the diameter of the CBD: the wider group (Group-W, CBD diameter≥8 mm, n=36) and the slender group (Group-S, CBD diameter<8 mm, n=19). The clinical data were compared and analysed.

**Results:**

No significant difference in the mean operative time, mean hospital stay or mean total charge was observed. Bile leakage occurred in 5.26%(1/19) of patients in Group-S, which accounted for only 1.81% (1/55) of total patients. There were no severe complications or mortality in either group. No significant differences in the rate of stone recurrence were observed. No biliary stricture occurred during follow-up.

**Conclusions:**

PDC may not be limited by the slender CBD (<8 mm), and the proper selection of patients and the standard surgical procedure are particularly important.

## INTRODUCTION

Acute cholangitis(AC) is an acute inflammation of the biliary tract caused mostly by common bile duct stones (CBDSs), which are present in approximately 10-15% of patients with symptomatic cholelithiasis^1^, and as a result, single-stage surgical treatment of both CBDSs and gallstones is required in many clinical cases. Laparoscopic common bile duct exploration (LCDBE) combined with laparoscopic cholecystectomy (LC) and endoscopic retrograde cholangiopancreatography (ERCP) combined with LC are two commonly used surgical options.^2^ The two approaches were reported to be comparable in terms of complication rate, mortality, and postoperative quality of life.^3^,^4^ Compared with ERCP, LCBDE has a lower incidence of duodenal perforation, bleeding and postoperative pancreatitis and has obvious advantages in saving the function of the Oddi sphincter, shortening the hospitalization duration and reducing the total cost .^4^ It is believed that LCBDE+LC can treat choledocholithiasis and cholecystolithiasis simultaneously in one operation and is advantageous in that simultaneous application can preserve the function of Oddi’s sphincter and reduce the incidence of long-term complications, which is especially important for young patients.^5^ The efficacy and safety of emergency LCBDE for the management of nonsevere cholangitis have been well established.^6^,^7^

Traditionally, LCBDE followed by T-tube drainage is a safe surgical procedure that is widely used for bile duct drainage and bile duct stone removal. However, T-tube drainage-related complications, such as drainage site pain, electrolyte disturbance, retrograde infection and unplanned extubation, are noteworthy.^8^,^9^ With the development of laparoscopic suturing techniques, the efficacy and safety of PDC for the management of nonsevere acute cholangitis are well documented.^10^,^11^

As mentioned in several previous studies, primary closure of the CBD incision was usually only performed if the diameter of the CBD was more than or equal to 8 mm.^12^-^15^ This idea was also supported by a singlearm metaanalysis and systematic review.^16^ However, all these reports were based on operations performed in patients with a CBD diameter of no less than 8 mm. There have been no studies on the perioperative outcomes or long-term follow-up of PDC in patients with a slender CBD (<8 mm). With the improvement of surgical skills, we tried PDC in patients with nonsevere acute cholangitis with a slender CBD (<8 mm) and received encouraging results.

A sufficient sample of patients were reviewed and followed up for more than 10 years. The clinical data were compared and analysed in this study, and the incidences of biliary stricture and stone recurrence were observed through long-term follow-up. The aim of the present study was to evaluate whether the efficacy and safety of PDC was limited to patients with a wider CBD (≥8 mm).

## METHODS

This was a retrospective study. Fifty-five patients who underwent PDC after LCBDE admitted to Taicang Affiliated Hospital of Soochow University (The First People’s Hospital of Taicang) from March 2012 to June 2019 were divided into the wider group (n=36) and the slender group (n=19) according to the diameter of the CBD. Patients’ clinical characteristics and health status were retrospectively obtained from the hospital information system. A group of experts evaluated comorbidities in accordance with expert consensus and guidelines.

**Fig.1:**
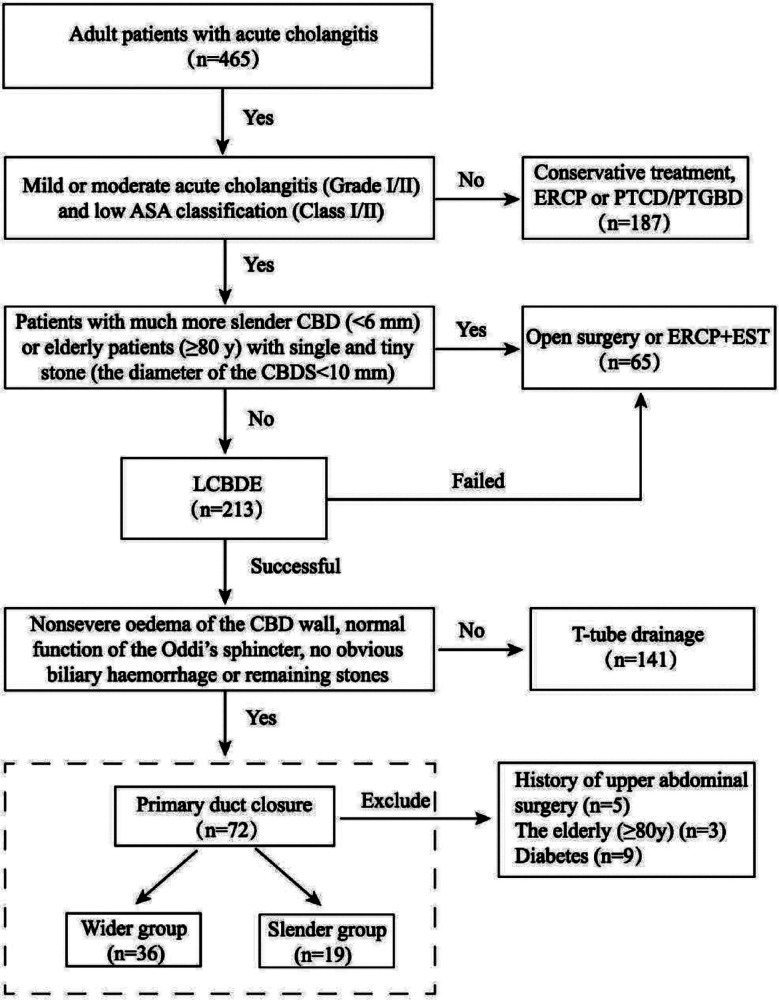
Study flowchart: A total of 55 patients enrolled in our s tudy. PTCD: percutaneous transhepatic cholangial drainage; PTGBD: percutaneous cholecystostomy for gallbladder drainage.

We screened a total of 465 patients with AC of whom 213 patients underwent LCBDE successfully from March 2012 to June 2019. Finally, 55 patients (23 males and 32 females) underwent PDC after LCBDE were enrolled in the study and were divided into two groups according to the diameter of the CBD: the slender group (<8 mm) and the wider group (≥8 mm). Perioperative and long-term follow-up data for patients are summarized (Fig.1).

### Ethical Approval:

The study was approved by the Institutional Ethics Committee of Taicang Affiliated Hospital of Soochow University (The First People’s Hospital of Taicang) (No.:2023-ky-029; Date: September 16, 2023), and written informed consent was obtained from all participants.

### Inclusion criteria:

- Adult patients (≥18 years) with a low ASA classification (Class-I or II).
- Mild or moderate acute cholangitis (Grade-I or II) and who underwent PDC after LCBDE.

### Exclusion criteria:

- Elderly patients (≥80 years).
- Patients with a history of upper abdominal surgery (including cholecystectomy, ERCP and EST) or diabetes.

Acute cholangitis(AC) was diagnosed based on the clinical symptoms and auxiliary examinations that are described in the Tokyo Guidelines (TG18).^16^ All patients were graded according to the American Society of Anaesthesiologists’ physical status (ASA) classification before surgery, and the severity of AC was graded according to the clinical symptoms and laboratory data described in the Tokyo Guidelines. In our centre, the management of acute cholangitis is based on the updated Tokyo Guidelines^17^, and a flow diagram for the management of acute cholangitis is shown in Fig.1.

### Surgical procedure:

The operation was performed using the conventional four-hole method. The main operating trocar (12 mm) for choledochoscopy was placed in the upper abdomen 3-cm under the xiphoid. The operation was started with dissection of Calot’s triangle. The gallbladder was removed after the cystic artery and cystic duct were clipped and cut. Then, a longitudinal choledochotomy of approximately 10 mm was made with sharp scissors after the CBD was clearly exposed and defined. A choledochoscope (OLYMPUS, CHF-V) was inserted for CBD exploration, and CBDSs were removed by a stone basket (WECARE, WSL-2040-L-Y). For patients with remaining large or fixed stones after unsuccessful removal attempts with stone baskets, a holmium laser (Accu-Tech, ACU-H20A) can be used to crush the stones.

**Fig.2:**
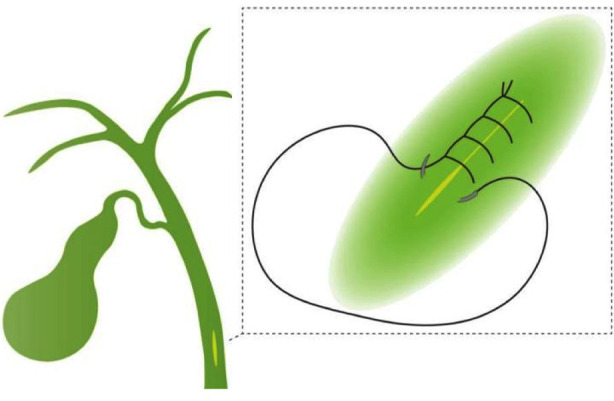
***Schematic illustration of PDC in our centre:*** The common bile duct is closed using a continuous overand-over locking approach. The distance between each needle is approximately 2mm, and the entry and exit points are approximately 1mm from the incisal edge of the common bile duct.

After that, choledochoscopy can be performed repeatedly to confirm the clearance of the biliary tract and the function of Oddi’s sphincter. For patients with nonsevere oedema of the CBD wall, normal function of Oddi’s sphincter, no obvious biliary haemorrhage or remaining stones, the CBD was primarily closed with monofilament synthetic absorbable 5-0 sutures (Ethicon Inc., PDS® II) using a continuous over-and-over locking fashion (Fig.2). Finally, the gallbladder and the stones were retrieved with a plastic fetch bag, and a negative pressure silicone drainage tube was routinely placed in the subhepatic space right beside the CBD. Intraoperative blood loss was calculated by the total amount of fluid aspirated minus the amount of intraoperative rinse fluid.

### Outcome definitions and follow-up:

The diameter of the CBD and stones were confirmed and measured by magnetic resonance cholangiopancreatography (MRCP) in all patients. Complications were defined as obvious abnormal indices within 30 days of surgery, and the severity of complications was graded according to the ClavienDindo classification.^18^ Bile leakage was defined as bilirubin concentration in the drain fluid at least three times the serum bilirubin concentration on or after postoperative day three or bile peritonitis that required active therapeutic or surgical intervention.^19^

The first and second follow-up examinations were conducted at one and three months after surgery in the outpatient clinic. After that, the patients were contacted by phone every 3-6 months. Patients were interviewed about their general conditions, diets, and surgery-related discomforts. Imaging tests, if necessary, were performed to screen for adverse events and detect possible biliary stricture and stone recurrence. Stone recurrence was defined as the presence of CBDSs diagnosed by MRCP or CT after complete stone removal. Biliary stricture was defined as repeated fever, jaundice, and abnormal liver function tests combined with an obviously narrow of common bile duct proved by MRCP. Follow-up time was defined as the time from discharge to the most recent visit or phone call. Loss to follow-up, death and the appearance of biliary stenosis were all considered endpoints, and this study ended with a final phone call in June 2023.

### Statistical analysis:

All data analyses were performed using SPSS, version 23.0 (SPSS, Inc., Chicago, IL, USA). The confidence interval was 95%. Categorical variables were analysed by the χ^2^ test or Fisher’s exact test. Continuous variables were expressed as the mean±standard deviation (SD), and comparisons of data were performed with the independent samples t test. P<0.05 was considered significant.

## RESULTS

A total of 55 patients were included in our study and were divided into two groups according to the diameter of the CBD. Thirty-six patients with a diameter of the CBD greater than or equal to 8 mm were included in Group-W, and the other 19 patients were included in Group-S. The wider group had a mean diameter of 11.6±3.2 mm (range 8.0-20.9), and the slender group had a mean diameter of 7.0±0.6 mm (range 6.0-7.8). Demographics and clinical characteristics of the included patients are summarized in Table-I.

**Table-I:** Demographics and clinical characteristics.

	Group-W (n=36)	Group-S (n=19)	P
Age (year)	53.44±16.56	44.53±16.08	0.061
***Sex [n (%)]***			0.544
Male	14(38.89)	9(47.37)	
Female	22(61.11)	10(52.63)	
***ASA classification [n (%)]***			0.592
I	24(66.67)	14(73.68)	
II	12(33.33)	5(26.32)	
***Preoperative liver function***			
TB (μmol/L)	33.53±33.16	34.61±29.75	0.906
ALT (U/L)	168.35±173.48	233.28±235.59	0.250
AST (U/L)	209.47±321.80	239.02±320.55	0.747
GGT (U/L)	280.03±239.91	273.64±319.73	0.934
***Severity Grade [n (%)]***			0.165
I	33(91.66)	18(94.74)	
II	3(8.34)	1(5.26)	
*The diameter of the CBD (mm)	11.58±3.19	6.96±0.60	<0.001

ASA classification: American Society of Anaesthesiologists Physical Status; TB: total bilirubin; ALT: alanine aminotransferase; AST: aspartate aminotransferase; GGT: γ-glutamyltransferase;

*: The diameter of CBD in images of MRCP.

The mean age of the patients was 53.4±16.6 years (range 22-78) in Group-W and 51.32±17.71 years (range 24-76) in Group-S, which was not significantly different (P=0.061). Sex (P=0.544) and the ASA classifications (P=0.592) were also not significantly different between the two groups. The preoperative liver function and indicators of infection severity were separately recorded and not significantly different.

The perioperative outcomes are reviewed in Table-II. The mean operating time was not significantly different between Group-W and Group-S (102.14±22.14 minutes for Group-W vs. 96.58±19.11 minutes for Group-S, P=0.36). Postoperative blood tests were carried out three days after the operation. Liver function (TB, ALT, AST and GGT) was not significantly different between the two groups. The hospitalization expenses (17470.92±3627.61 CNY for Group-W vs. 16438.39±3643.90 CNY for Group-S, P=0.32) and postoperative hospitalization (7.1±1.2 days for Group-W vs. 6.7±1.0 days for Group-S, P=0.22) were also not significantly different between the two groups.

**Table-II:** Perioperative outcomes.

	W group (n=36)	S group (n=19)	P
Operating time (min)	102.14±22.14	96.58±19.11	0.358
***Postoperative liver function***			
TB (μmol/L)	21.99±14.42	22.4±9.74	0.901
ALT (U/L)	87.50±113.70	103.16±66.20	0.584
AST (U/L)	38.75±46.13	41.79±26.83	0.793
GGT (U/L)	223.33±224.74	153.69±127.82	0.219
Hospitalization expenses (CNY)	17470.92±3627.61	16438.39±3643.90	0.321
Postoperative hospitalization (day)	7.08±1.20	6.68±1.00	0.222
***Complications [n (%)]***			
Bile leakage	0	1(5.26)	1
Retained CBDs	0	0	1
Abdominal infection	1(2.78)	0	1
Biliary hemorrhage	0	0	1
Clavin-Dindo classification (classI/II) [n]	0/1	1/0	1
Mortality [n (%)]	0	0	1

TB: Total bilirubin; ALT: Alanine aminotransferase; AST: Aspartate aminotransferase; GGT: γ-glutamyltransferase.

One patient (2.78%) in Group-W had a residual abdominal infection, which was cured by antibiotic treatment. One patient (5.26%) in Group-S suffered from bile leakage, which was cured by unobstructed drainage and symptomatic treatments. All complications were graded as Class I or II according to the ClavienDindo classification, and there were no serious complications or mortalities.

Long-term follow-up of patients was performed in our study, and the information is reviewed in Table-III. Five patients (9.09%) were lost to follow-up after the first follow-up examination because they refused to cooperate with the phone interview. In addition, the remaining 50 patients (91.91%) maintained good contact, with a median follow-up period of 97.00±30.36 months (range 45-135) in Group-W and 88.53±23.66 months (range 52-132) in Group-S. There was no significant difference in the follow-up time (P=0.295) or the rate of follow-up to date (P=0.473) between the two groups.

**Table-III:** Outcomes of long-term follow-up.

	W group (n=36)	S group (n=19)	P
Follow-up to date [n (%)]	32(88.89%)	18(94.74%)	0.473
Follow-up time (months)	97.00±30.36	88.53±23.66	0.295
Stone recurrence [n (%)]	2(5.56%)	1(5.26%)	1
Early recurrence (≤2 years)	1(2.78%)	0	
Late recurrence (>2 years)	1(2.78%)	1(5.26%)	
Management of stone recurrence [n (%)]			1
ERCP	1(2.78%)	0	
Re-operation	1(2.78%)	1(5.26%)	
Biliary stricture [n (%)]	0	0	1

In Group-W, one recurrence was detected within two years after surgery, and another recurrence was detected more than two years after surgery. Moreover, only one late recurrence was detected in Group-S more than two years after surgery. There were no significant differences in the recurrence rate between the two groups. One patient was treated with ERCP in Group-W because of a small and single recurrent stone, and the rest underwent reoperation (LCBDE with PDC). There was no case of stone recurrence until the end of this study. No manifestations of biliary stricture were detected in either group.

## DISCUSSION

In this study, our results suggest that CBD with a diameter less than 8-mm may not be a contraindication for PDC. By grouping, we found for the first time that PDC in a patient with a slender CBD did not result in a higher incidence of complications, stone recurrence or biliary stricture. This was different from the theory that the safe diameter of the CBD for PDC was more than or equal to 8-mm.^15^ It was gradually believed that PDC might be able to replace T-tube drainage and might become the trend in the future.^20^ However, there has been no consensus on the safe diameter of CBD for PDC. According to their clinical experience, most researchers agree that a wider CBD (≥8 mm) is safe for primary closure, but it is impossible to verify the source of this safe diameter, and there is a lack of evidence on the safe diameter of CBD for PDC. Deng *et al*.^15^ reported that the clinical indication for PDC after LCBDE should include having a safe diameter (diameter of CBD≥8 mm). However, a singlearm study was unable to demonstrate that a slender CBD (<8 mm) was unsafe for PDC. There have been no specialized studies on the safety of PDC in patients with a slender CBD (<8 mm). Therefore, this does not mean that PDC is risky for patients with a CBD diameter that is less than 8 mm. We believe that there may be no significant differences between the two groups in terms of surgical efficacy and safety, and a slender CBD may have no obvious disadvantage for PDC.

In addition, we observed some differences from previous studies in terms of bile leakage and stone recurrence. Bile leakage occurred in only 1.82% (1/55) of the patients in our study, which was significantly lower than that in previous study.^10^ One of 19 patients (5.26%) in Group-S had bile leakage, which was similar to a previous study about “the safe diameter of PDC (≥8 mm)”.^15^ According to the outcomes of the follow-up, there were two cases of stone recurrence (5.56%) in Group-W and one case (5.26%) in Group-S, which was slightly higher than a retrospective clinical study with a median follow-up of 18 months.^10^ We found that stone recurrence might occur more than 24 months after surgery, so a short-term follow-up may not be sufficient, and the actual rate of stone recurrence might be much higher. Moreover, there was no biliary stricture in either group which was similar to another retrospective study.^21^

According to the results of this study, we believe that PDC performed in patients with a slender CBD may not cause a significant increase in the risk of bile leakage, stone recurrence or biliary stricture, which may be due to the details of our surgical procedure. The key points of our surgical procedure are making a sharp longitudinal incision on the CBD with sharp scissors instead of an electrotome and closing the incision with a 5-0 PDS II suture in a continuous over-and-over locking manner. This surgical procedure and suture method may minimize the formation of scars, and PDS II sutures can effectively prevent bile leakage through the needle holes and reduces the chance of stone recurrence. Moreover, better closure and haemostasis can be achieved with as few tissues as possible if the sutures are placed in a continuous over-and-over locking manner. Hence, bile leakage and biliary haemorrhage can be effectively prevented, and the width of the CBD can be greatly preserved. The outcomes of the present study indirectly supported this hypothesis. In this study, the incidence of bile leakage (1.82%) was quite low, and biliary haemorrhage and biliary stricture were not observed. Although our surgical procedure requires a higher level of suturing skills and a longer operating time, it is highly recommended, especially for patients with a slender CBD. It has become the standard surgical procedure for PDC in our centre.

The only case of bile leakage in this study occurred in Group-S. Does it mean that a slender CBD is more prone to bile leakage? Following a logistic regression analysis, Liu *et al*.^14^ reported that bile leakage might occur more frequently in patients with a slender CBD (<10 mm). In their study, bile leakage occurred in 11.3% of the patients who underwent primary closure following LBCDE and more frequently in patients with a slender CBD (<10 mm). Hua *et al*.^12^ found that a bile duct width less than 8 mm and retained CBDSs are risk factors for bile leakage. In the first study, despite high recommendations, neither the continuous over-and-over locking suture technique nor the PDS II suture line was used, which may have contributed to the increase in the overall rate of bile leakage. There were only four patients with a slender CBD (<8 mm) in the second study, and not much meaningful statistical power could be obtained. Unlike these studies, the rate of bile leakage was not as high in our study. One of 19 patients (5.26%) in Group-S had bile leakage, which was similar to a previous study about “the safe diameter of PDC (≥8 mm)”.^15^ We believe that the diameter of the CBD as a risk factor for bile leakage needs to be further studied in multiple centres.

It is worth noting that cases with much a more slender CBD (<6 mm) were excluded in our study. Since our choledochoscope has a diameter of 5.85 mm (insertable portion), in our experience it is difficult to perform choledochoscopy for biliary exploration and stone extraction after LCBDE when the diameter of the CBD is less than 6 mm. In our centre, LCBDE is only performed if the diameter of the CBD is greater than or equal to 6 mm. Therefore, the diameter of the CBD was between 6-8 mm (excluding 8 mm) in Group-S, and patients with a much more slender CBD (<6 mm) were usually advised to undergo open surgery or ERCP+EST. With the help of finer choledochoscopes and more refined surgical skills, the safe diameter of PDC will be further explored in future.

### Limitations:

It includes small sample size. In future, we need to enlarge the sample size. At the same time a multicenter study is needed to further evaluate the safe diameter of the CBD for LCBDE with PDC.

## CONCLUSIONS

Common bile duct(CBD) with a diameter less than 8 mm may not be a contraindication for PDC. Proper patient selection and standard surgical procedures are critical.

### Authors’ Contributions:

**DS** and **YL:** Concept, design, carried out the studies, data collection, drafted the manuscript

**PC Haibin X Hongxing X:** Performed the statistical analysis and participated in its design and did Review.

All authors read and approved the final manuscript and are responsible and accountable for the accuracy or integrity of the work.
